# Role of the Filifactor alocis Hypothetical Protein FA519 in Oxidative Stress Resistance

**DOI:** 10.1128/Spectrum.01212-21

**Published:** 2021-11-10

**Authors:** Ezinne Aja, Arunima Mishra, Yuetan Dou, Hansel M. Fletcher

**Affiliations:** a Division of Microbiology and Molecular Genetics, Department of Basic Sciences, School of Medicine, Loma Linda University, Loma Linda, California, USA; University of Illinois at Urbana Champaign

**Keywords:** biofilm, *Filifactor alocis*, oxidative stress, periodontal disease, peroxidase

## Abstract

In the periodontal pocket, there is a direct correlation between environmental conditions, the dynamic oral microbial flora, and disease. The relative abundance of several newly recognized microbial species in the oral microenvironment has raised questions on their impact on disease development. One such organism, Filifactor alocis, is significant to the pathogenic biofilm structure. Moreover, its pathogenic characteristics are highlighted by its ability to survive in the oxidative-stress microenvironment of the periodontal pocket and alter the microbial community dynamics. There is a gap in our understanding of its mechanism(s) of oxidative stress resistance and impact on pathogenicity. Several proteins, including HMPRFF0389-00519 (FA519), were observed in high abundance in F. alocis during coinfection of epithelial cells with Porphyromonas gingivalis W83. Bioinformatics analysis shows that FA519 contains a “Cys-X-X-Cys zinc ribbon domain” which could be involved in DNA binding and oxidative stress resistance. We have characterized FA519 to elucidate its roles in the oxidative stress resistance and virulence of *F. alocis*. Compared to the wild-type strain, the *F. alocis* isogenic gene deletion mutant, FLL1013 (Δ*FA519*::*ermF*), showed significantly reduced sensitivity to hydrogen peroxide and nitric oxide-induced stress. The ability to form biofilm and adhere to and invade gingival epithelial cells was also reduced in the isogenic mutant. The recombinant FA519 protein was shown to protect DNA from Fenton-mediated damage with an intrinsic ability to reduce hydrogen peroxide and disulfide bonds. Collectively, these results suggest that *FA519* is involved in oxidative stress resistance and can modulate important virulence attributes in *F. alocis*.

**IMPORTANCE** Filifactor alocis is an emerging member of the periodontal community and is now proposed to be a diagnostic indicator of periodontal disease. However, due to the lack of genetic tools available to study this organism, not much is known about its virulence attributes. The mechanism(s) of oxidative stress resistance in *F. alocis* is unknown. Therefore, identifying the adaptive mechanisms utilized by *F. alocis* to survive in the oxidative stress environment of the periodontal pocket would lead to understanding its virulence regulation, which could help develop novel therapeutic treatments to combat the effects of periodontal disease. This study is focused on the characterization of FA519, a hypothetical protein in *F. alocis*, as a multifunctional protein that plays an important role in the reactive oxygen species-detoxification pathway. Collectively, our results suggest that FA519 is involved in oxidative stress resistance and can modulate important virulence attributes in *F. alocis*.

## INTRODUCTION

Overcoming or adapting to oxidative stress is an important element in the survival of microorganisms in the periodontal pocket (1). This oxidative stress environment is usually generated via reactive oxygen species (ROS), such as the superoxide anion (O_2_^.-^), hydroxyl anion (OH^−^), hydrogen peroxide (H_2_O_2_), nitric oxide (NO), and peroxynitrite (ONOO^−^), which are O_2_ derivatives and byproducts of aerobic metabolism (2) or the host immune response (3, 4). Bacteria must have mechanisms that detoxify the ROS to prevent damage to cellular components, such as nucleic acids, membrane lipids, and proteins (5).

Filifactor alocis is classified as a slow-growing, Gram-positive, asaccharolytic, obligate anaerobic rod bacterium that is an important periodontal pathogen (6–9). *F. alocis*, in cooccurrence with a dysbiotic polymicrobial community, can trigger periodontitis, which is a multifaceted chronic inflammatory disease that affects the supporting structures of the teeth (10, 11). In comparison with the other traditional periodontal pathogens, including members of the “red complex” bacteria (Porphyromonas gingivalis, Tannerella forsythia, and Treponema denticola), the high incidence of *F. alocis* in the periodontal pocket, compared with its absence in healthy individuals or those who are periodontitis resistant, has highlighted its importance in the etiology of the disease (7, 12, 13). Additionally, studies have established links between periodontal disease and systemic diseases such as pneumonia, cardiovascular disease, rheumatoid arthritis, Alzheimer’s disease, preterm-low-birth weight delivery, and some cancers (11, 14–17).

Previous reports have demonstrated that *F. alocis* has virulence properties that are necessary for host cell invasion, survival/persistence, and pathogenesis (18–20). Additionally, *F. alocis* is relatively more resistant to H_2_O_2_-induced oxidative stress compared to P. gingivalis (18). Moreover, under H_2_O_2_-induced stress conditions, the survival of P. gingivalis is increased more than 4-fold when grown in coculture with *F. alocis* (H.M. Fletcher, personal communication). Collectively, these observations suggest that *F. alocis* may have an innate ability to detoxify the local inflammatory microenvironment of the periodontal pocket. The genome of *F. alocis* shows that it is missing catalase activity as well as other conventional mechanism(s), including ROS scavengers that could enable its survival in the periodontal pocket (21). Recently, the characterization of the first antioxidant enzyme “superoxide reductase” (SOR) in *F. alocis* was shown to play an important role in the defense against superoxide radicals, air exposure, and H_2_O_2_-induced oxidative stress (21).

There continues to be a gap in our understanding of the roles of other gene(s) and/or mechanism(s) of oxidative stress resistance in *F. alocis* (21). In coculture with P. gingivalis W83, there was an upregulation of *F. alocis* proteins, including several that are known in other bacteria to be involved in oxidative stress resistance (such as iron-sulfur cluster proteins, superoxide reductase, thioredoxin family proteins) as well as several hypothetical proteins of unknown function (22). One such hypothetical protein, FA519, which is encoded by the gene *HMPREF0389_00519* (designated *FA519*), was also shown to be upregulated during coinfection of epithelial cells with P. gingivalis W83 (22). Bioinformatics analysis shows that FA519 contains a Cys-X-X-Cys zinc ribbon domain (X: any amino acid) which, as shown in other bacteria, could be involved in DNA binding and oxidative stress resistance (reviewed in reference 23). In this study, we characterized FA519 to elucidate its roles in oxidative stress resistance and virulence of *F. alocis*. We explored the sensitivity of an *F. alocis* isogenic mutant (Δ*FA519*) to hydrogen peroxide and nitric oxide-induced stress. The ability of the isogenic mutant to form biofilm and adhere to and invade gingival epithelial cells was compared to that of the wild-type *F. alocis* strain. The purified recombinant FA519 protein was shown to protect DNA from Fenton-mediated damage with an intrinsic ability to reduce hydrogen peroxide and disulfide bonds. Collectively, our results suggest that *FA519* is involved in oxidative stress resistance and can modulate important virulence attributes in *F. alocis*. A mechanistic role for FA519 in oxidative stress resistance in *F. alocis* and its potential impact on pathogenicity are discussed.

## RESULTS

### *F. alocis* HMPREF0389_00519 encodes a hypothetical protein with a putative nucleic acid-binding domain and zinc ribbon domain.

**FIG 1:**
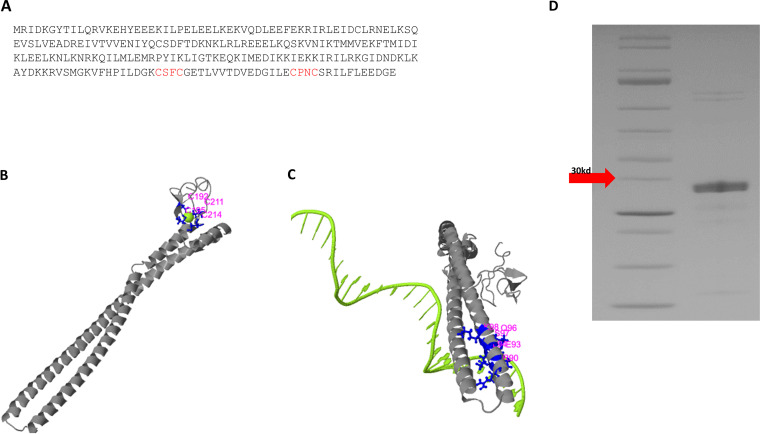
*In silico* analysis of FA519. FA519 is a hypothetical protein. (A) Amino acid sequence of FA519. Cys-X-X-Cys motif is indicated in red. FA519 is predicted to have (B) a C4-type-Zn ribbon at the C terminus and (C) a long coiled-coil DNA-binding domain at the N terminus. (D) SDS-PAGE gel showing rFA519 with molecular weight ∼30 kDa.

The genome of *F. alocis* carries the *HMPREF0389_00519* gene, which is 678 bp in length and encodes a 225-amino-acid hypothetical protein (https://www.ncbi.nlm.nih.gov/protein/291166556) (Fig. 1A). Protein modeling using the I-TASSER software shows that the FA519 protein contains a putative zinc ribbon domain with two Cys-X-X-Cys motifs at amino acid positions 192 to 195 and 211 to 214 (Fig. 1B). There is a putative N-terminal DNA-binding domain (Fig. 1C). The recombinant FA519 (rFA519) was purified from Escherichia coli and resolved on SDS-PAGE as an ∼29.6× His tag fusion protein (Fig. 1D).

### *FA519* is upregulated under hydrogen peroxide-induced stress when *F. alocis* is in coculture with P. gingivalis.

**FIG 2:**
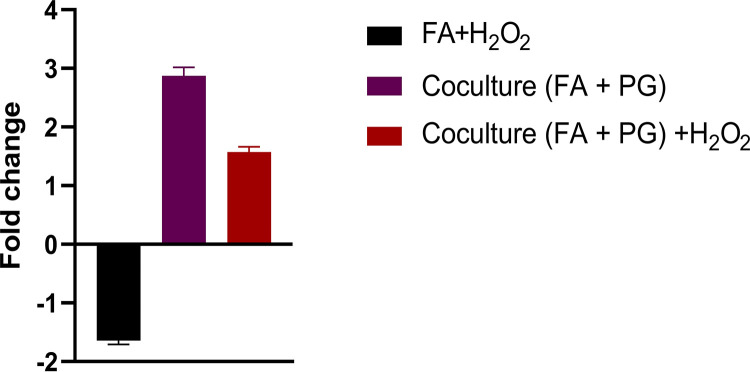
*FA519* is upregulated when *F. alocis* is in coculture with P. gingivalis. RT-qPCR analysis was carried out on *F. alocis* monoculture exposed to 0.25 mM H_2_O_2_ for 15 min, *F. alocis* in coculture with P. gingivalis, and *F. alocis* in coculture with P. gingivalis exposed to 0.25 mM H_2_O_2_ for 15 min.

A previous study analyzing the proteome during coinfection of epithelial cells with *F. alocis* and P. gingivalis has shown upregulation of several *F. alocis* proteins, including the hypothetical protein, *F. alocis* FA519, along with several other proteins known to be involved in oxidative stress resistance (24). To further evaluate the induction of *FA519* under oxidative stress conditions, *F. alocis* in coculture with P. gingivalis was exposed to 0.25 mM H_2_O_2_ for 15 min and a whole-transcriptome analysis was done by transcriptome sequencing (RNA-Seq). In addition to *FA519*, which showed a 2.2-fold increase, approximately 47% (817 genes) of the *F. alocis* genome displayed altered expression (fold change of ≥2.0, *P* value of ≤0.05; see Table S1 in the supplemental material). There were 374 upregulated genes and 443 downregulated genes in *F. alocis*. It is noteworthy that 38 of the genes upregulated encode proteins that carry Cys-X-X-Cys motifs, and 7 of these proteins are annotated as hypothetical with unknown function (Table S2). To confirm the RNA-Seq expression profile, we carried out real-time quantitative PCR to confirm if *FA519* was upregulated. As shown in Figure 2, the *FA519* gene was downregulated when *F. alocis* was exposed to 0.25 mM H_2_O_2_ for 15 min in the absence of P. gingivalis. This is in contrast to its upregulation in coculture with P. gingivalis exposed to the same concentration of H_2_O_2_ for 15 min. It is also noteworthy that the *FA519* gene was upregulated in *F. alocis* in coculture with P. gingivalis in the absence of H_2_O_2_-induced stress.

### FLL1013 showed significant sensitivity to H_2_O_2_- and NO-induced stress compared to the wild-type strain.

**FIG 3:**
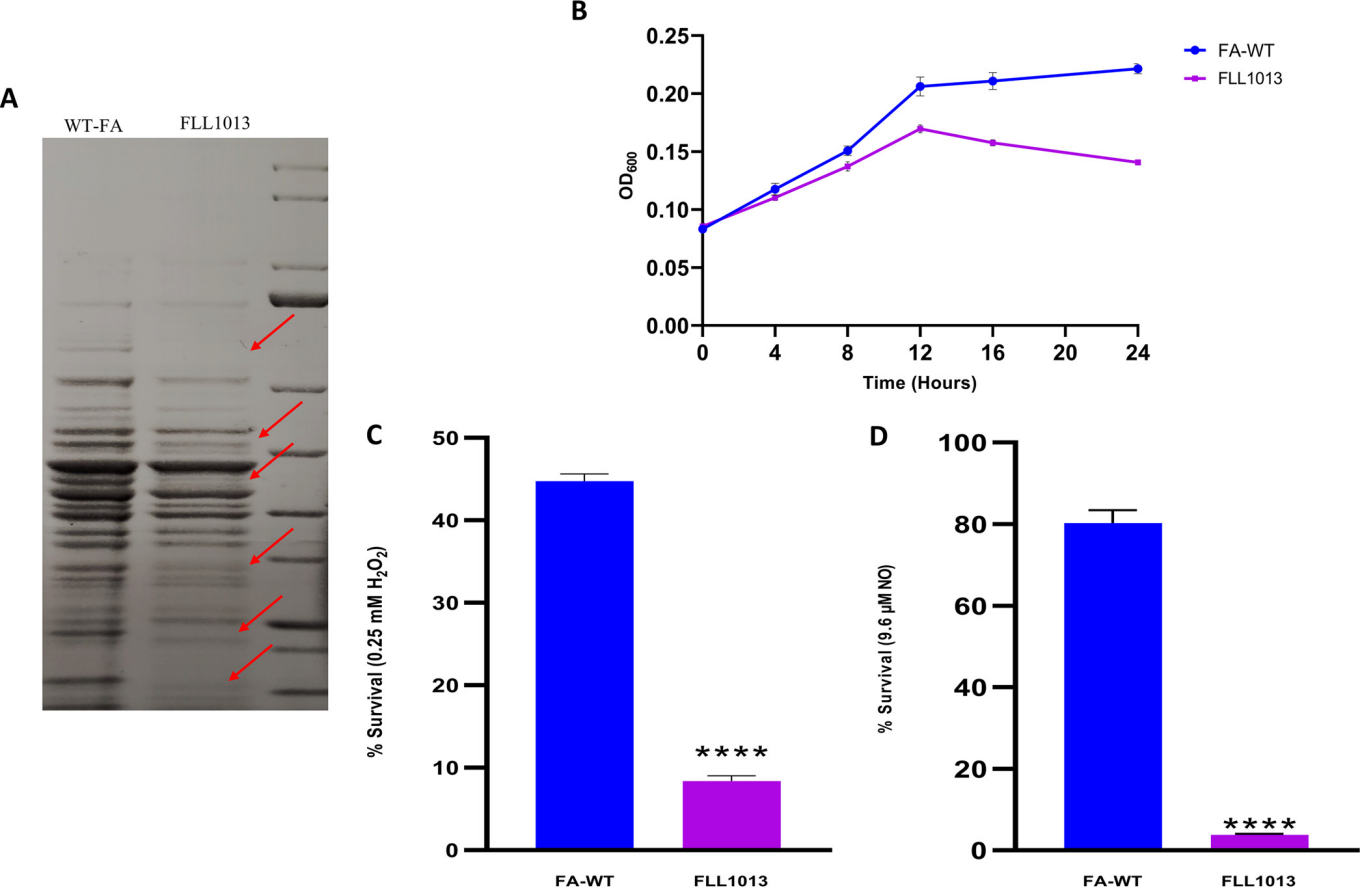
FLL1013 is more sensitive to oxidative stress than FA-WT. (A) Comparison of protein expression in the wild-type strain compared to FLL1013. Red arrows indicate putative differences in protein expression of *F. alocis* wild-type strain compared to that of FLL1013. (B) FLL1013 grows slower than *F. alocis* wild type (FA-WT). The growth of the bacterial strains was determined spectrophotometrically. The OD_600_ was measured at 3-h intervals over a 24-h period. Each experiment was done in triplicate. The error bars show standard deviation. (C and D) FLL1013 is more sensitive to H_2_O_2_- and NO-induced stress compared to the wild-type strain. Briefly, overnight bacterial strains, wild-type strain and FLL1013, were grown in BHI and then diluted in BHI medium without cysteine to a starting concentration of ∼0.05 OD_600_. (C) 0.25 mM H_2_O_2_ or (D) 9.6 μM DEANONOATE (NO donor) was added to the cultures and incubated anaerobically at 37°C for 1 h. The percentage of surviving bacteria was calculated relative to the number of bacteria without exposure to stress agents. Statistical analysis was performed using two-tailed unpaired Student’s *t* test (****, *P* < 0.0001 versus control).

To further evaluate a functional role for FA519 in oxidative stress protection, an isogenic mutant of *F. alocis* deficient in this gene was constructed by allelic-exchange mutagenesis. The *FA519*-deficient mutants were selected on Brain Heart Infusion (BHI) agar plates carrying erythromycin. Deletion of the *FA519* gene in the erythromycin-resistant isogenic mutants was confirmed by colony PCR and DNA sequencing (data not shown). To compare their phenotypic properties with those of the wild-type *F. alocis* strain, all erythromycin-resistant colonies were plated onto BHI blood agar plates. Similarly to the wild-type strain, they all displayed small, translucent, beige colonies (data not shown). One mutant, designated *F. alocis* FLL1013 (Δ*FA519*::*ermF*), was randomly chosen for further studies. In contrast to that of the wild type, the protein profile of the isogenic mutant was altered (Fig. 3A). Compared to the wild-type strain, FLL1013 had a lower growth rate with a lower cell density at optical density at 600 nm (OD_600_) (Fig. 3B). FLL1013 also showed increased sensitivity to H_2_O_2_ (Fig. 3C) and NO (Fig. 3D) compared to the wild type. Taken together, these results suggest that the *FA519* gene may play a role in tolerance to H_2_O_2_- and NO-induced stress.

### FLL1013 does not make biofilm compared to the wild-type strain.

**FIG 4:**
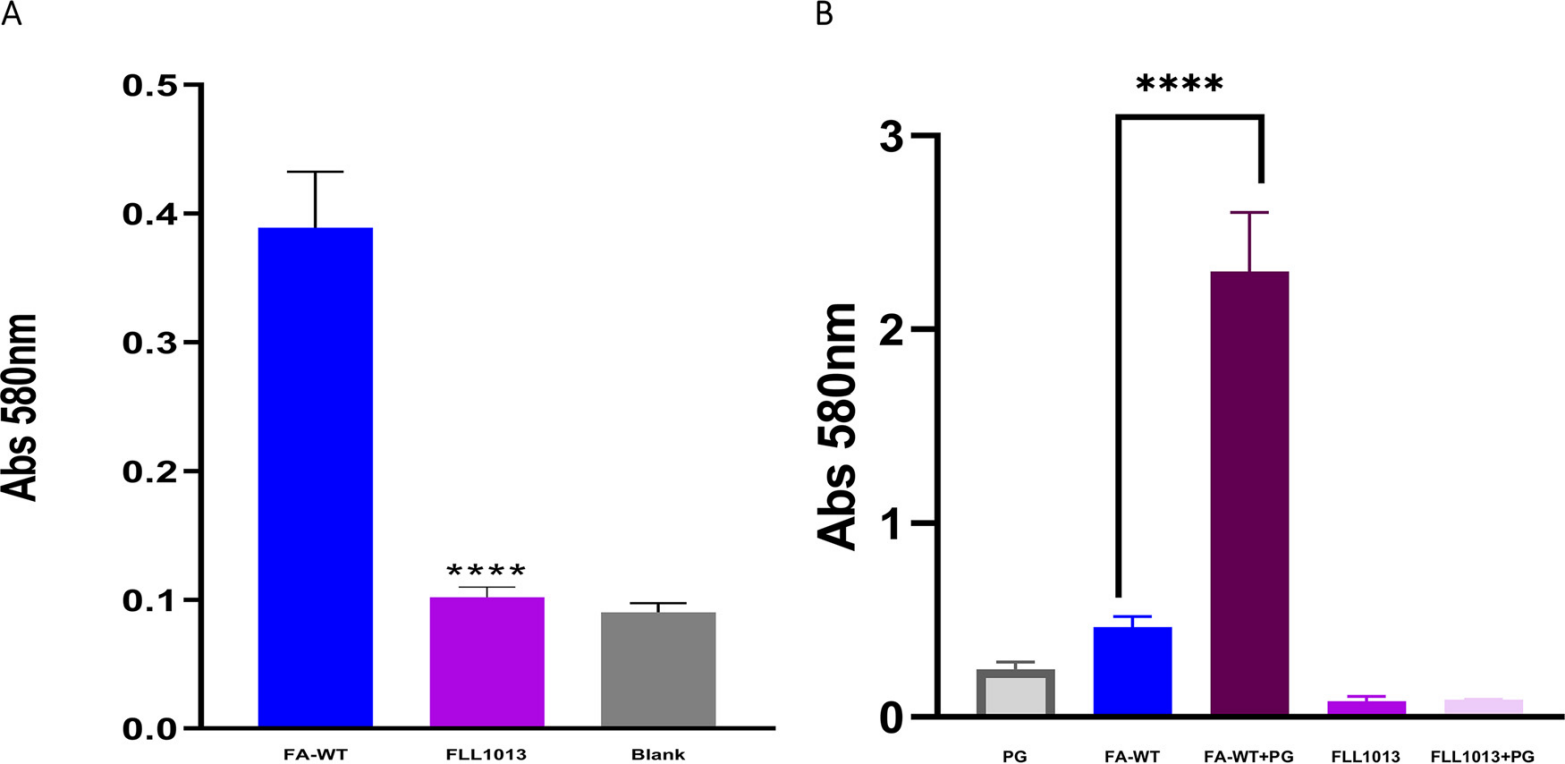
Role of *FA519* in *in vitro* biofilm formation. (A) *F. alocis* strains (wild-type and FLL1013) and (B) coculture strains (*F. alocis* strains and *PG33277*) were grown in 96-well plates in BHI broth for 48 h. Bacterial biofilms were stained with 0.5% crystal violet and quantitated by measuring the absorbance at 580 nm. BHI broth was used as blank. The values presented here are the means of four independent experiments. Error bars represent the standard deviations from the means. Statistical analysis was performed using two-tailed unpaired Student’s *t* test (****, *P* < 0.0001 versus control).

A biofilm assay was used to determine the ability of *F. alocis* wild-type strain and FLL1013 to form mono species and dual species biofilm with *PG33277*. As shown in Figure 4A, the biofilm-forming capacity of the FLL1013 was significantly reduced compared to that of the wild-type strain. In coculture with *PG33277*, the biofilm-forming capacity was significantly increased. However, FLL1013 in the presence of *PG33277* was unable to form biofilm (Fig. 4B). These results show that *FA519* can affect the *in vitro* formation of *F. alocis* biofilms.

### *FA519* plays a role in the survival of *F. alocis* in host cells.

**FIG 5:**
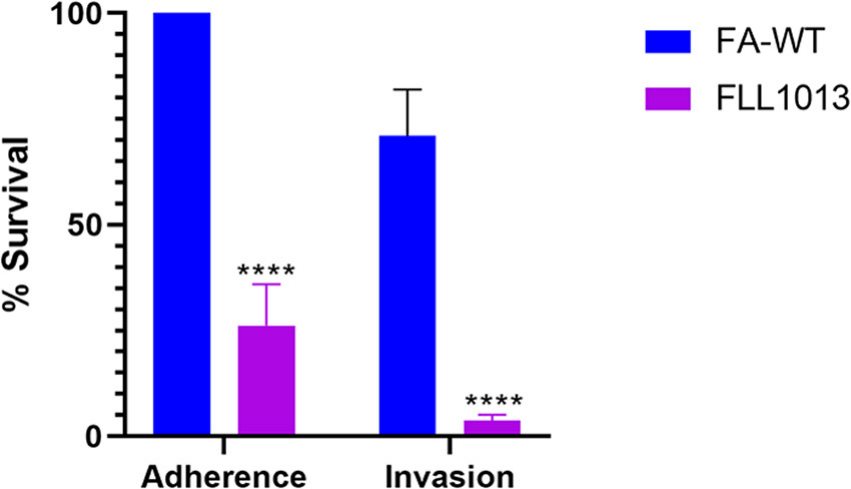
Adherence and invasion of *F. alocis* wild-type strain and FLL1013 mutant in TIGK cells. Epithelial cells (10^5^ cells) were infected with 10^7^ bacteria (MOI: 100) for 1 h in an anaerobic chamber. The percent adhesion and invasion were calculated, and data are presented as percentage for the mutant compared to that of the wild-type strain, which was normalized to 100%. Experiments were carried out in three independent repeats in triplicates. Error bars represent the standard deviations from the means. Statistical analysis was performed using two-tailed unpaired Student’s *t* test (****, *P* < 0.0001 versus control).

Adherence and invasion of gingival epithelial cells by periodontopathogens are important steps in the pathogenesis process. Therefore, the adhesion and invasion abilities of *F. alocis* wild-type strain and FLL1013 in telomerase-immortalized gingival keratinocytes (TIGKs) were determined. As shown in Figure 5, following 1 h of incubation, there was an ∼74% reduction in the adhesion abilities of FLL1013 to the wild-type strain (normalized to 100%). FLL1013 also had an ∼67% decrease in invasiveness compared to that of the wild-type strain. These results suggest that *FA519* plays a role in the survival of *F. alocis* in host cells.

### rFA519 binds Zn.

**FIG 6:**
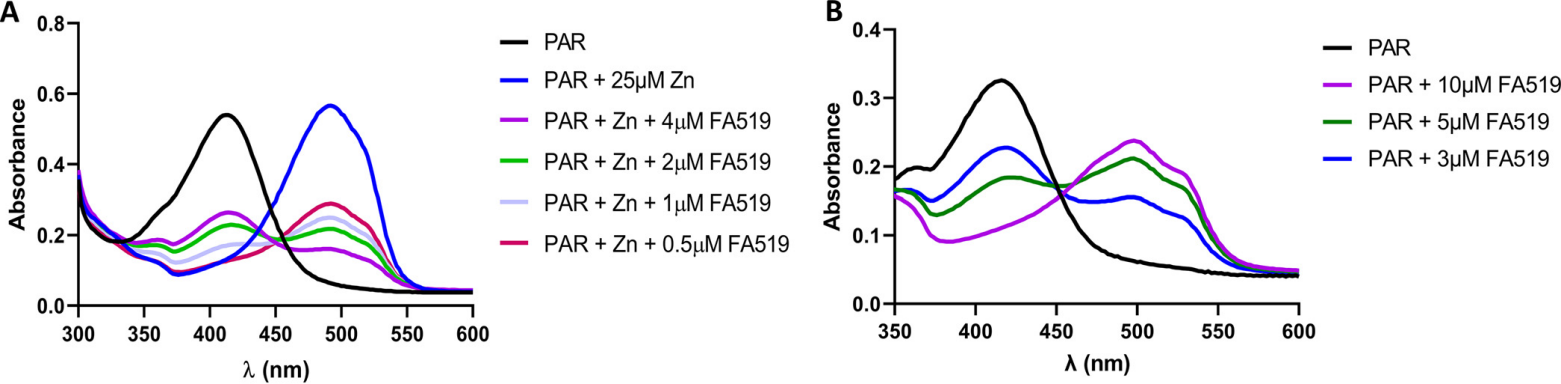
rFA519 binds zinc. Free PAR exhibits a peak absorbance at approximately 410 nm, which shifts to 500 nm upon Zn binding. (A) Addition of increasing concentration of rFA519 to PAR-Zn shows an observed shift in spectrum from ∼500 nm to ∼410 nm, which indicates zinc binding. (B) Addition of purified rFA519 to PAR shows an increased absorbance at 500 nm, which suggests that rFA519 may be purified bound to Zn.

FA519 is predicted to be a zinc ribbon domain protein. To determine if the FA519 binds Zn, we performed a PAR [4-(2-pyridylazo)-resorcinol] assay to ascertain the presence of Zn in the recombinant protein as well as to measure Zn binding. This assay is based on the principle that PAR exhibits a peak absorbance at approximately 410 nm in its free form, while this peak shifts to approximately 500 nm upon Zn binding. Addition of a Zn-binding protein to PAR-Zn leads to a reduction in the peak at 500 nm and an increase in the peak at 410 nm. When increasing concentrations of purified rFA519 were added to PAR-Zn, the Zn-bound peak at 500 nm decreased while the peak at 410 nm increased, establishing that rFA519 binds to Zn *in vitro* (Fig. 6A). Scanning of the spectrum of increasing concentrations of rFA519 with PAR shows an increase at 500 nm, which suggests that the purified protein could be bound to zinc (Fig. 6B).

### rFA519 protects DNA from Fenton-mediated DNA damage.

**FIG 7:**
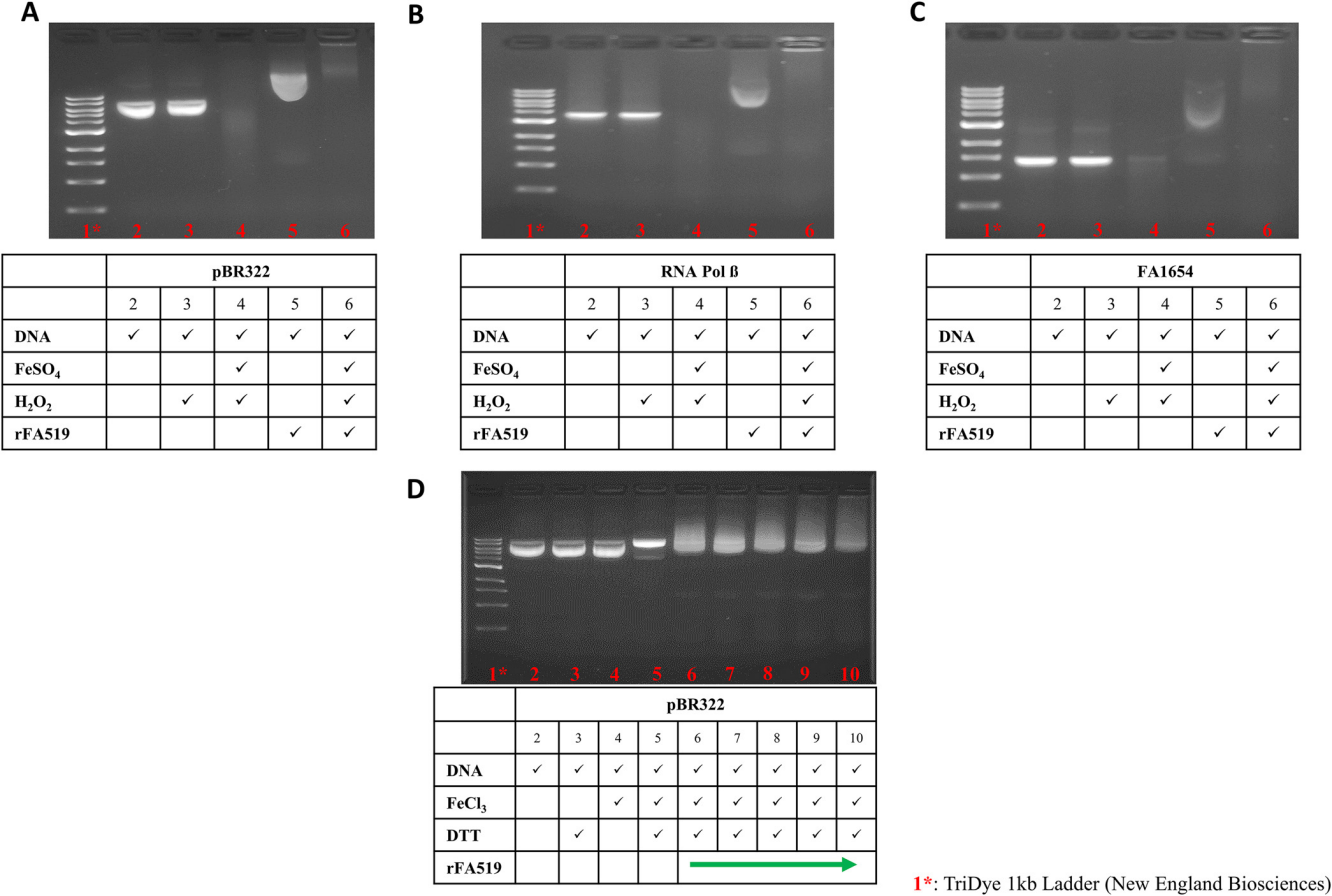
rFA519 protects DNA from oxidative damage *in vitro.* The 1% agarose gel shown was loaded with various reaction components and indicated amounts of rFA519. (A to C) Lane 1, TriDye 1kb Ladder; lane 2, DNA alone; lane 3, DNA plus H_2_O_2_; lane 4, DNA plus full Fenton reaction (Fe^2+^ and H_2_O_2_) without rFA519; lane 5, DNA plus 10 μM rFA519; lane 6, DNA plus full Fenton reaction plus 10 μM rFA519. Lane 5 (A to C) shows that 10 μM rFA519 abolishes the effects of the Fenton reaction and results in the retardation of the DNA. (A) pBR322 plasmid DNA. (B) RNA polymerase β subunit. (C) FA1654. (D) Lane 1, TriDye 1kb Ladder; lane 2, DNA alone; lane 3, DNA plus DTT; lane 4, DNA plus FeCl_3_; lane 5, DNA plus FeCl_3_ plus DTT; lane 6 to 10, DNA plus FeCl_3_ plus DTT plus rFA519 (2, 10, 15, 20, and 25 μM), respectively. Lanes 6 to 8 show that rFA519 prevented the MCO-mediated nicking of the supercoiled pBR322 plasmid DNA.

The increased sensitivity of FLL1013 to H_2_O_2_- and NO-induced stress in addition to a putative nucleic acid binding function of rFA519 could suggest a DNA protection role in the presence of Fe^2+^ and H_2_O_2_. Therefore, the ability of FA519 to protect DNA against Fenton-mediated damage was assessed. Plasmid DNA (pBR322) or PCR-amplified DNA fragments (DNAJ, RNA polymerase β subunit, and FA1654) were mixed with rFA519 and incubated for 15 min prior to the addition of FeSO_4_ (50 μM) and H_2_O_2_ (2 μl; 880 mM stock). In the presence of the rFA519 protein, there was DNA binding and reduced DNA damage under oxidative stress-induced conditions (Fig. 7A to C, lane 6). The migration of the DNA fragments was retarded in the reaction with the rFA519 protein under normal conditions (Fig. 7A to C, lane 5). Taken together, this suggests that FA519 nonspecifically binds and protects DNA from Fenton-mediated DNA damage.

### rFA519 protects DNA from MCO.

Reactive oxygen species generated from the metal-catalyzed oxidation (MCO) system nick the supercoiled form of plasmid DNA into the linear form. An antioxidant protein can inhibit this process (25). We tested the ability of rFA519 to protect supercoiled DNA from nicking in the presence of FeCl_3_ and dithiothreitol (DTT). The shift in gel mobility of pBR322 as it was converted from the supercoiled to the nicked form indicated the extent of DNA damage (Fig. 7D, lane 5). In the absence of the rFA519, the reactive oxygen species produced in the MCO system caused nicking of the supercoiled pBR322 DNA (Fig. 7D, lane 5). The addition of rFA519, in increasing concentrations (2, 10, 15, 20, and 25 μM), to the MCO system prevented nicking of the supercoiled DNA by the reactive oxygen species generated in the assay (Fig. 7D, lanes 6 to 10). Smearing of the gel and a slight electrophoretic shift in the presence of rFA519 suggest interaction between the plasmid DNA and rFA519 (Fig. 7D, lanes 6 to 10).

### Antioxidant activity of rFA519.

**FIG 8:**
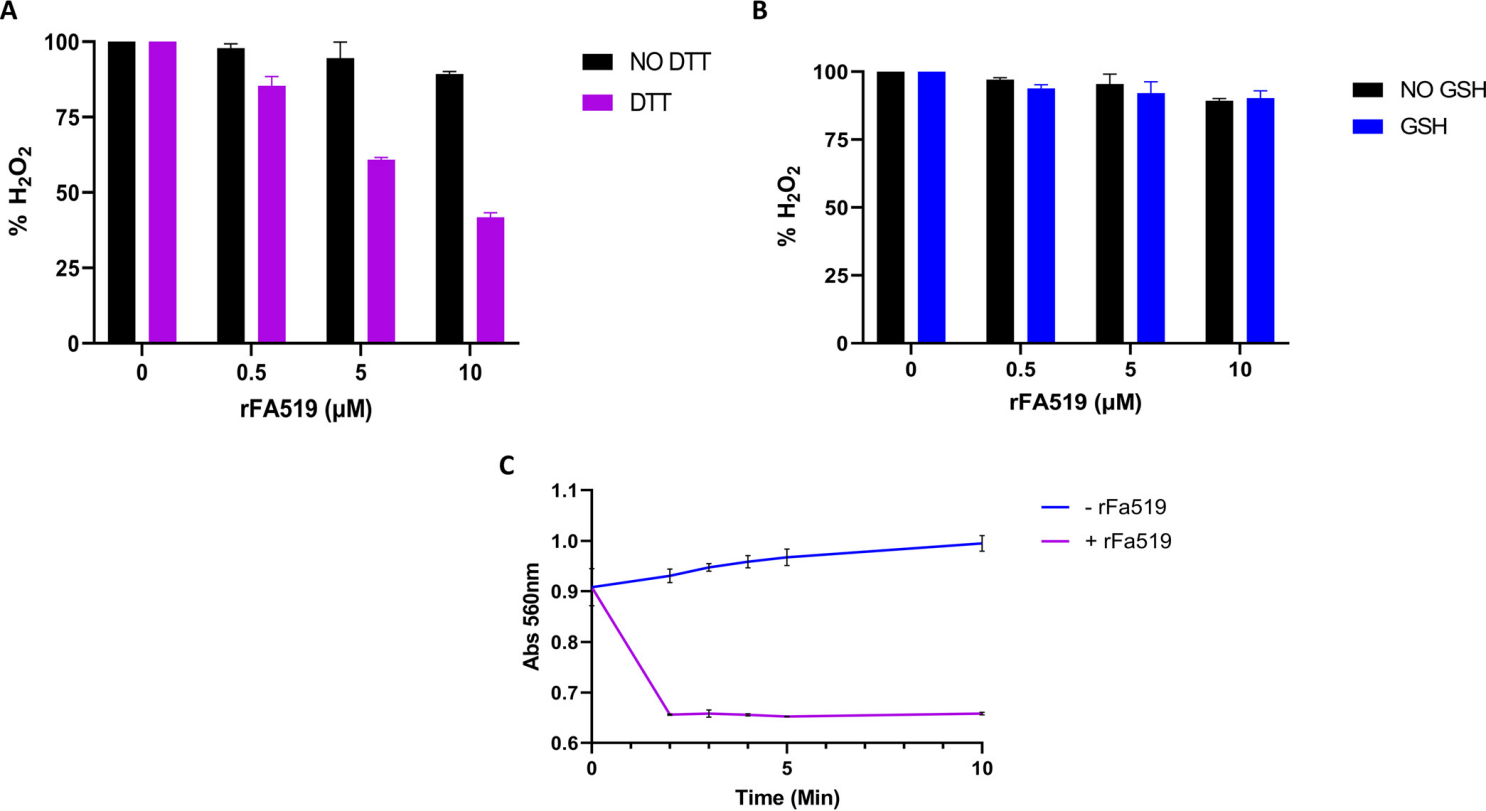
Antioxidant activity of rFA519. Removal of H_2_O_2_ by rFA519 with (A) DTT or (B) GSH. Increasing concentrations of rFA519 (0.5, 5, and 10 μM) were preincubated with or without 5 mM dithiothreitol (DTT) or 5 mM glutathione (GSH) and H_2_O_2_. The reaction was stopped by addition of trichloroacetic acid, and Fe(NH_4_)_2_(SO_4_)_2_ and KSCN were added to react with the remaining H_2_O_2_ to produce the ferrithiocyanate complex measured by colorimetry at 475 nm. H_2_O_2_ concentration has been normalized to 100%. (C) Peroxidase activity was also confirmed using the ferrous xylenol orange method. Concentrations of 10 μM rFA519, 1 mM DTT, and 100 μM H_2_O_2_ were incubated in potassium phosphate buffer (pH 7.0). At 0, 2, 3, 4, and 5 min, the assay mixture was added to xylenol orange solution. The decrease in H_2_O_2_ levels was determined spectrophotometrically by measuring absorbance at 560 nm. Control experiments included reactions with and without rFA519.

Since rFA519 was able to protect DNA from Fenton and MCO-mediated damage, the antioxidant activity of rFA519 that may result in the breakdown of H_2_O_2_ was evaluated using the ferrithiocyanate system. As shown in Figure 8A, the rFA519 protein was able to degrade H_2_O_2_ in the presence of DTT. This degradation activity was protein concentration dependent. In the presence of glutathione (GSH), the rFA519 protein failed to significantly degrade H_2_O_2_ (Fig. 8B). In the presence of xylenol orange, the degradation activity of rFA519 was further confirmed with the direct detection of the H_2_O_2_ levels. The rFA519 protein was able to degrade H_2_O_2_ in the presence of DTT indicated by a reduction in absorbance at 560 nm (Fig. 8C). Taken together, the data suggest that FA519 may have peroxidase activity. Moreover, the presence of two intramolecular disulfide bonds, which require two-thiol donor reducers, such as DTT, may support the inability of GSH (a one-thiol donor) to reduce the oxidized form of rFA519, likely regenerating its enzymatic activity.

### The antioxidant activity of rFA519 is linked to thioredoxin.

**FIG 9:**
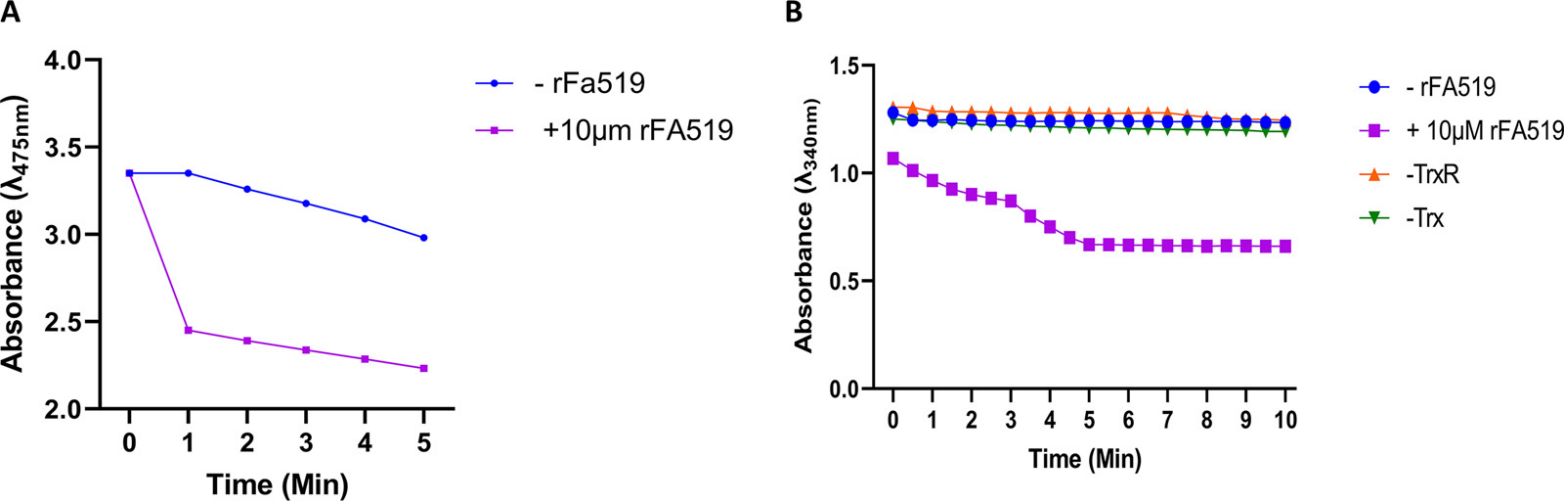
Thioredoxin-linked antioxidant activity of rFA519. (A) Peroxidase activity of rFA519 using the thioredoxin system as a reductant. Using the ferrithiocyanate system, rFA519 was able to reduce H_2_O_2_, indicated by a reduction in absorbance at 475 nm. (B) NADPH-linked peroxidase activity of rFA519. NADPH oxidation was monitored by the decrease of *A*_340_ in 50 mM HEPES-NaOH (pH 7.0) containing 0.375 mM NADPH, 20 μg thioredoxin, 6.25 μg thioredoxin reductase, 10 μM rFA519, and 1 mM H_2_O_2_. Complete system (purple square), minus rFA519 (blue circle), or thioredoxin (green upside-down triangle), or thioredoxin reductase (orange triangle).

In the presence of DTT, a thiol-containing electron donor, rFA519, was able to detoxify H_2_O_2_. Therefore, we investigated the ability of an enzymatic thiol-regenerating system (Trx, TrxR, and NADPH) to activate the antioxidant activity of rFA519. In the presence of Trx, TrxR, and NADPH, we observed a decrease in absorbance at 480 nm using the ferrithiocyanate system (Fig. 9A), suggesting that rFA519 may utilize thioredoxin as a reductant. To further elucidate the peroxidase activity of rFA519, we monitored oxidation of NADPH. In this system, rFA519 uses H_2_O_2_ as a substrate and the Trx-TrxR system transfers electrons from NADPH to rFA519. We found that rFA519 catalyzed the reduction of H_2_O_2_ with its activity dependent on the presence of the Trx-TrxR recycling system indicated by a decrease in absorption at 340 nm. NADPH oxidation required all components and was negligible in the absence of any one of them (Fig. 9B).

### rFA519 possesses oxidoreductase activity.

**FIG 10:**
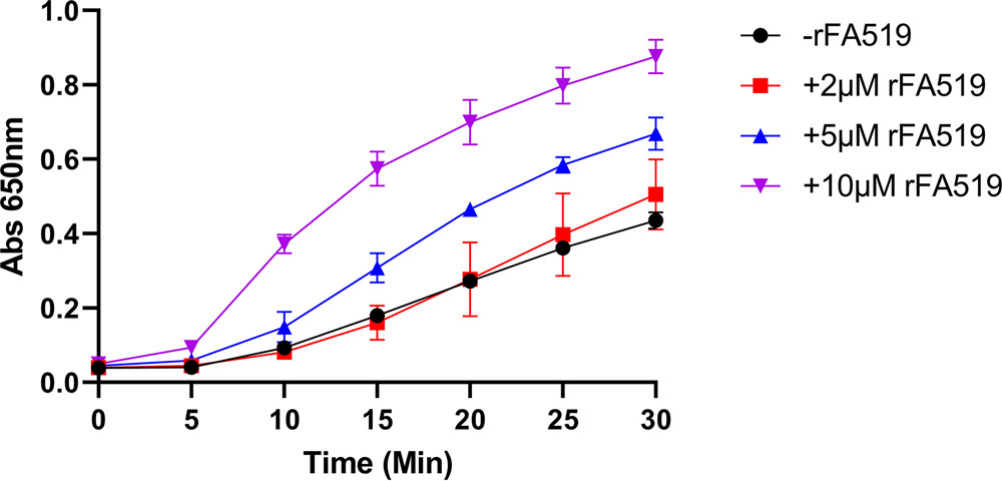
Oxidoreductase activity of rFA519. Reaction mixtures contained 150 μM insulin and increased concentrations of rFA519 (2 μM, red square; 5 μM, blue triangle; 10 μM, purple upside-down triangle) in 0.1 M potassium phosphate buffer (pH 7.0) and 2 mM EDTA. Reactions were started by adding 1 mM DTT. After thorough mixing, the optical density at 650 nm was recorded every 5 min. Control reactions were done without rFA519 (black circle).

Several proteins that carry Cys-X-X-Cys motifs are known to have oxidoreductase characteristics (26, 27). The oxidoreductase activity of rFA519 was evaluated *in vitro* by determining its ability to reduce insulin. Insulin contains two intramolecular disulfide bonds that connect the A and B chains, and reduction of these disulfide bonds causes the precipitation of the B chain, which can be monitored by following the increase of turbidity at 650 nm. As shown in Figure 10, there was an increase in turbidity when insulin was incubated in the presence of various concentrations of the rFA519 protein. The data suggest that the activity is concentration dependent and that rFA519 may likely function as an oxidoreductase.

## DISCUSSION

Periodontal disease, the sixth most common infectious disease, affects over 65 million people in the United States (28). As an inflammatory disease, the resulting tissue destruction and concomitant induction of oxidative stress can arise from an excessive inflammatory response to the biofilm-associated bacterial community with the accompanying release of reactive oxygen species (ROS), such as H_2_O_2_ and superoxide radicals from neutrophils (reviewed in reference 29). There is emerging evidence for a vital role of *F. alocis* in the adaptation of the microbial community to oxidative stress in the inflammatory microenvironment of the periodontal pocket. Its pathogenic characteristics are highlighted by its ability to survive in the oxidative-stress-rich environment of the periodontal pocket and significantly alter the microbial community dynamics by forming biofilms and interacting with several oral bacteria (reviewed in reference 30). Collectively, these observations suggest that *F. alocis* may likely have the inherent ability to induce adaptation and/or detoxify the oxidative stress environment of the periodontal pocket. Currently, the genes that may play a role in oxidative-stress-resistance mechanism(s) in *F. alocis* are unknown or implied at best. In this study, we have functionally characterized the *F. alocis HMPREF0389_00519* gene that encodes a hypothetical protein with putative nucleic acid-binding and zinc ribbon domains.

The *F. alocis* FA519 protein carries a zinc ribbon domain with two Cys-X-X-Cys motifs at the C terminus. Many bacterial enzymes and regulatory proteins that can perform a wide range of functions in various cellular processes also carry the conserved zinc-containing redox Cys-X-X-Cys motifs (31). This signature cysteine zinc ribbon can be associated with ROS-mediated signaling and an oxidative stress response as well as zinc binding and stabilization of protein domains that are essential for function (23, 31–33). Our studies have indicated that FA519 possesses zinc-binding ability and its purification under native conditions may retain zinc. The functional role of zinc, including its *in vivo* significance, is unclear and remains under investigation in the laboratory. Similar to these observations, the *F. alocis* FA519 protein in our studies likely plays a significant role in the defense mechanism(s) to oxidative stress. The *F. alocis* FA519-deficient isogenic mutant showed significant sensitivity to H_2_O_2_- and NO-induced stress. The relative significance is further supported by its modulation to H_2_O_2_-induced stress. It is noteworthy that its highest response occurs in coculture with P. gingivalis. Although the expression of the *FA519* gene in *F. alocis* appears to require its interaction with P. gingivalis, its level of expression was reduced in the presence of H_2_O_2_-induced oxidative stress in coculture with P. gingivalis. However, *FA519* gene expression was significantly downregulated when *F. alocis* alone was exposed to H_2_O_2_-induced oxidative stress. This suggests that the microbial community dynamic is important for its expression and protective functional role. It is also likely that under oxidative stress, other pathways might be induced with less reliance on *FA519*; therefore, we cannot rule out the possibility that *FA519* may have a different functional activity under oxidative stress compared to other conditions. Multiple other genes that are modulated in *F. alocis* when in coculture with P. gingivalis can likely have an impact on its survival in the oxidative stress environment of the periodontal pocket. Several of these genes known to be involved in oxidative stress pathways in other bacteria, such as rubredoxin, rubrerythrin, thioredoxin family proteins and their reductases, several disulfide reductases, and other hypothetical proteins, were upregulated in *F. alocis.* Several of these proteins, including the hypothetical proteins, carry Cys-X-X-Cys motifs. While a current mechanism is unknown, we cannot rule out the impact of this synergistic effect on oxidative stress resistance of the microbial community in the periodontal pocket. The relative resistance of *F. alocis* to oxidative stress, in addition to its ability to enhance the survival of P. gingivalis under those environmental conditions, further supports an important role for *F. alocis* in microbial community dynamics in the periodontal pocket (24).

*F. alocis* is seen in significantly high numbers in the periodontal pockets of periodontitis patients, likely because of its ability to form biofilms (9). *F. alocis* has been shown to form *in vivo* biofilms and colocalizes with different bacteria species, including P. gingivalis, Prevotella intermedia, Aggregatibacter actinomycetemcomitans, T. denticola, T. forsythia, and Fusobacterium nucleatum (9). H_2_O_2_ is reported to reach millimolar concentrations in oral bacterial biofilms (5, 34). Therefore, to successfully be a member of the oral biofilm, *F. alocis* needs to possess the ability to counter the oxidative effects of the environment and express the appropriate surface receptors to facilitate its interaction with the microbial community. Consistent with a previous report (18), *F. alocis* in coculture with *PG33277*, a known biofilm-forming strain of P. gingivalis, showed a significantly increased biofilm-forming capacity. In contrast, that biofilm-forming capacity was absent in *F. alocis* FLL1013 in the presence of *PG33277*. These observations could support an alteration in the surface structure(s) in *F*. *alocis* FLL1013. This would be consistent with the inability of *F. alocis* FLL1013 to adhere to and invade gingival keratinocytes. Moreover, these results suggest a role for the *FA519* gene in the ability of *F. alocis* to survive in host cells. The effect of FA519 on the expression of specific surface receptors in *F. alocis* is under further investigation in the laboratory.

The FA519 protein could likely be part of a complex regulatory network with multifunctional properties. The rFA519 protein was able to protect DNA from Fenton-mediated damage and was able to bind to the DNA likely by forming large DNA-protein complexes. This ability to form large complexes with DNA is characteristic of DNA protection from starvation (Dps) proteins and could be a unique feature of FA519. E. coli and Campylobacter jejuni Dps both bind DNA when there is an accumulation of H_2_O_2_ (35). Zinc ribbon proteins are also known to be able to bind nucleic acids, proteins, and other small molecules (31). In the metal-catalyzed oxidation (MCO) system, ROS, particularly H_2_O_2_ and hydroxyl radicals, are generated in the presence of oxygen in a mixture of Fe^3+^ and DTT. These ROS result in single-strand DNA breaks (25). rFA519 prevented nicking of the supercoiled DNA by the ROS generated and showed an ability to bind DNA. The strongest DNA-binding ability of rFA519 was observed in the Fenton reaction. rFA519 was able to bind DNA in the absence of oxidative stress, and there was less binding under the MCO system. It is possible that exogenous sources of H_2_O_2_, such as those released from immune cells or other bacteria species, will lead to an increase in the DNA-binding abilities of rFA519. ROS generated by the MCO system might not be at a high enough level to activate the avid binding of FA519, or a separate mechanism of DNA might be modulated based on the source of ROS. Putative mechanisms of DNA binding and the possibility of different DNA-binding domains of FA519 may be involved and warrant further exploration. Overall, our results indicate that rFA519 binds DNA, which likely provides DNA protection from hydroxyl radicals. Also, its ability to modulate gene expression is unknown and awaits further investigation.

FA519 can detoxify H_2_O_2_, suggesting that it has peroxidase activity. As shown in our study, this activity was enhanced in the presence of a two-thiol reducing agent. This is consistent with the Cys-X-X-Cys motifs in FA519 that are expected to give rise to reduced disulfide bonds in the presence of DTT. Thus, to be functional under oxidative stress condition, it is likely that in *F. alocis*, the FA519 protein needs to be in a reduced state. This FA519 reduced state, under oxidative stress condition, can be generated by a thioredoxin system as demonstrated in our study. It is noteworthy that several oxidative-stress-related genes (including thioredoxin [Trx] family proteins) were upregulated in *F. alocis* in coculture with P. gingivalis. Their functional role as part of this redox cycle awaits further investigation in the laboratory. Moreover, we cannot rule out the involvement of other proteins that carry Cys-X-X-Cys motifs and are annotated as hypothetical proteins with unknown function.

Most currently characterized proteins with a catalytic Cys-X-X-Cys motif are members of the thioredoxin (Trx) and/or disulfide oxidoreductase families (36, 37). The rFA519 protein was found to catalyze the reduction of insulin in the presence of DTT, which suggests that it possesses some disulfide oxidoreductase activity. Similar proteins with the thioredoxin folds function as disulfide oxidoreductases and interact with a broad range of proteins either for electron transport in substrate reduction or for regulation of activity via thiol-redox control (38–40). Disulfide oxidoreductases may play important roles in the defense against oxidative stress (41, 42) by protecting damaged proteins from inactivation and prevent aggregates of misfolded protein by reduction of disulfide bonds or through their formation in properly folded substrate proteins (42).

**FIG 11:**
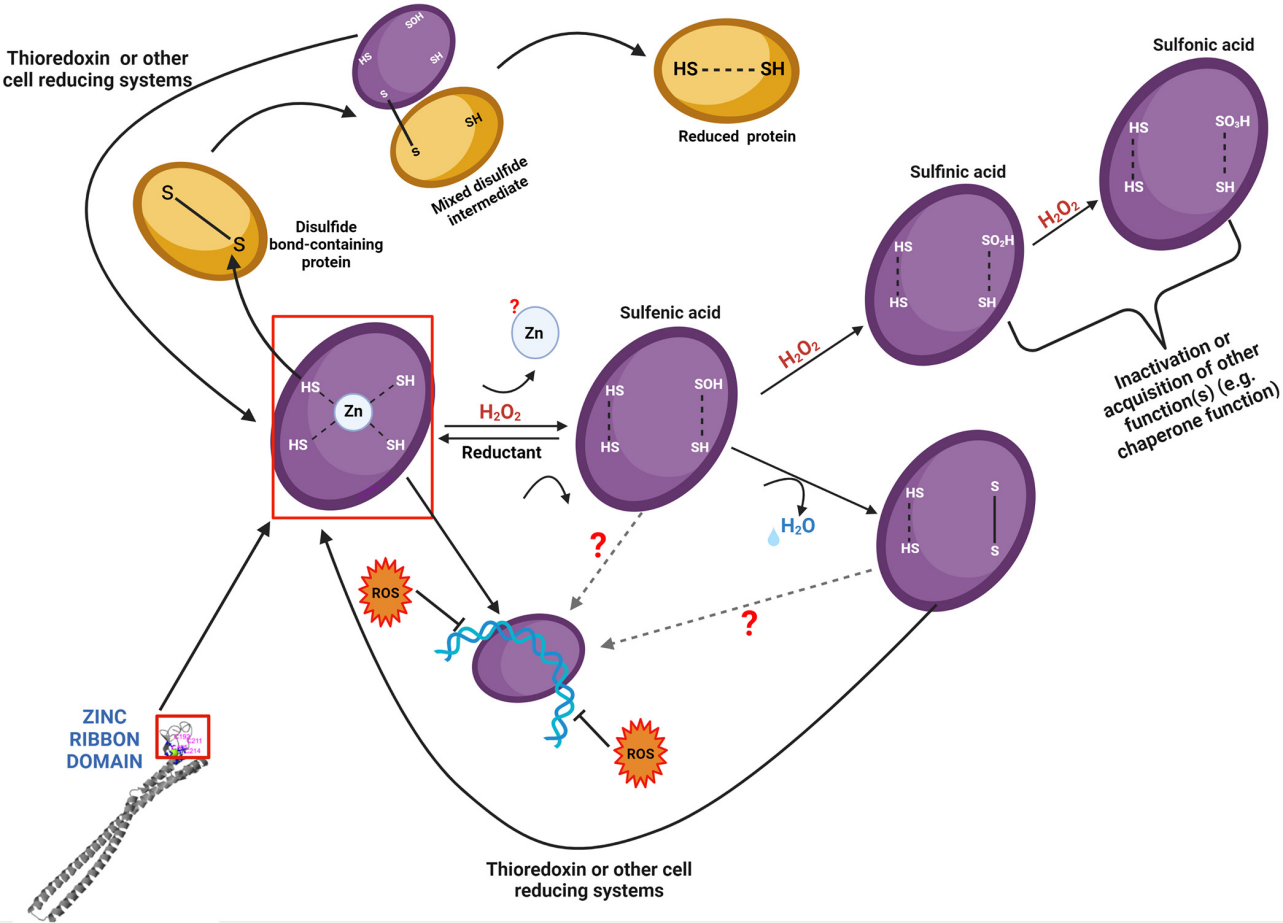
Proposed roles for FA519. This model schematically depicts proposed multifunctional roles for FA519. The FA519 utilizes conserved cysteines to reduce peroxides. The oxidation of cysteine thiol(s) in FA519 by ROS (such as H_2_O_2_) leads to the decomposition of H_2_O_2_ and may lead to the formation of highly reactive sulfenic acid. The formation of sulfenic acid is transient and likely reacts with another cysteine thiol in the protein to form a disulfide bond. The oxidation of the cysteine thiols is reversible and may be catalyzed by cell-reducing systems, such as the thioredoxin system. Further oxidation of the sulfenic acid to sulfinic acid and sulfonic acid may not be reversible *in vivo* and may render protein inactive or lead the acquisition of other functions. The role of zinc in FA519 remains yet to be determined but is likely dependent on its release in the oxidized state that may lead to conformational changes in the C-terminus that may affect the function of the protein. Our results show that FA519 binds DNA with an increased ability to bind and protect DNA from Fenton-mediated damage as well as metal-catalyzed oxidation. The exact conformation of FA519 needed to bind and protect DNA from ROS damage remains yet to be determined. FA519 may also function similarly to other Cys-X-X-Cys-containing proteins that are able to reduce disulfide bonds in substrate proteins. It is likely that a cysteine of the Cys-X-X-Cys motif attacks the disulfide bond on the substrate protein, leading to an intermediate mixed-disulfide complex. The other cysteine in the motif then reduces the mixed-disulfide complex, which releases a reduced substrate protein, and an oxidized FA519 that could be reduced again by cell-reducing systems such as the thioredoxin system. Experiments to validate this model and to determine which cysteine residues are involved in the specific mechanism proposed are ongoing.

Collectively, the data from this study suggest that the multifunctional *F. alocis* FA519, which may represent a novel class of thioredoxin family proteins, can protect DNA from Fenton-mediated damage with intrinsic peroxidase and disulfide oxidoreductase activities. The functional pathway(s) of this protein and its role in oxidative stress resistance in *F. alocis* may be part of a complex network with unknown regulatory components. For example, this raises questions on how the multiple functions of FA519 are coordinated and/or regulated. Are there other reducing pathways in *F. alocis* that are capable of activating FA519 under conditions of oxidative stress? How is the *FA519* gene regulated since *F. alocis* lacks the typical oxidative stress regulators like OxyR and SoxR? Many proteins utilize cysteine thiols to regulate their own activity or play key roles in maintaining cellular redox homeostasis (reviewed in references 37, 43, and 44). Enzymes such as alkyl hydroperoxide reductases, peroxiredoxins, and thiol-dependent peroxidases that have been shown to degrade H_2_O_2_ and organic peroxides employ cysteines as a redox sensor in regulating H_2_O_2_-mediated cell signaling and homeostasis (44–46). Because of its observed role and protective effect against ROS, it is crucial to understand the biochemical and physiological mechanisms utilized by *FA519* to confer resistance to oxidative stress in *F. alocis*. Our observations taken together support a model (Fig. 11) which shows that FA519 may function similarly to other antioxidant proteins where redox cycling of cysteine plays a role in detoxification of peroxides and reduction of disulfide bond-containing proteins. In a likely scenario, the *F. alocis* SOR, reported previously (21), reduces superoxide radicals to H_2_O_2._ A potential mechanism to eliminate the H_2_O_2_ may involve the FA519 protein in its reduced state. The mechanism(s) involved in the activation of the DNA-binding properties as well as the functional role of zinc binding/release is still unclear. Studies to further validate the unique role of *FA519* in modulating the response of *F. alocis* to the oxidative stress environment of the periodontal pocket are ongoing in the laboratory. The ability for FA519 to modulate important virulence attributes in *F. alocis* has significant clinical implications.

## MATERIALS AND METHODS

### Bioinformatics analysis.

Bioinformatics analysis was carried out to predict the domains present in *F. alocis* FA519 protein (HMPREF0389_00519). Amino acid sequence and information on the conserved domains were obtained from the National Center for Biotechnology Information (NCBI) database (NC_016630.1). Domain and structure predictions were carried out using the I-TASSER software (https://zhanglab.ccmb.med.umich.edu/I-TASSER/).

### Bacterial strains and growth conditions.

All bacterial strains and plasmids used in this study are listed in Table 1. Unless otherwise stated, the *F. alocis* strains were grown in Brain Heart Infusion (BHI) broth supplemented with vitamin K (0.5 μg/ml), hemin (5 μg/ml), cysteine (0.1%), and arginine (100 μM). *F. alocis* cultures were grown anaerobically and maintained in an anaerobic chamber (Coy Manufacturing) with 10% H_2_, 10% CO_2_, and 80% N_2_. E. coli strains were grown in Luria-Bertani (LB) medium with appropriate antibiotics. Growth rates were determined spectrophotometrically by assessing the optical density at 600 nm (OD_600_). Unless otherwise stated, all cultures were incubated at 37°C. Erythromycin concentration was 5 μg/ml (for *F. alocis*) and ampicillin concentration was 100 μg/ml (for E. coli).

**TABLE 1:** Bacterial strains and plasmids

Bacteria strains/plasmids	Description	Reference or source
*F. alocis* strains		
FA ATCC 35896	Wild-type strain	22
FLL1013	Δ*FA519*::*ermF*, an isogenic derivative of FA ATCC 35896	This study
P. gingivalis strains		
PG W83	Wild-type strain	57
PG ATCC 33277	Type strain	47
E. coli strains		
Top 10	Genotype: F- mcrA Δ(mrr-hsdRMS-mcrBC) Φ80lacZΔM15 ΔlacΧ74 recA1 araD139 Δ(araleu)7697 galU galK rpsL (StrR) endA1 nupG. Used for general cloning purposes.	Invitrogen
BL21(DE3)	Genotype: F- ompT hsdSB (r_B_− m_B_−) gal dcm rne131 (DE3). Used as protein expression strain.	Invitrogen
Plasmids		
pPROEX HTA	Plasmid containing Amp^R^, lacI^q^, and P_trc_	Invitrogen
pVA2198	Sp^r^, *ermF-ermAM*	50

### RNA-Seq library construction and sequencing.

RNA-Seq was carried out as described previously (47). Briefly, rRNA was removed from 2 μg of total RNA using a RiboMinus transcriptome isolation kit (Life Technologies, Carlsbad, CA). The first-strand and second-strand cDNA synthesis, the RNA-sequencing barcode ligation, and other steps of libraries’ construction were performed using a Nextflex RNA-Seq kit according to the protocol of the manufacturer (Bioo Scientific, Austin, TX). The PCR-amplified cDNA samples were sequenced using a NextSeq 550 sequencing system (Illumina Inc., San Diego, CA), with 75-bp reads and at least 10 million reads per sample.

### Real-time quantitative PCR.

RNA isolation and cDNA synthesis were performed as described previously (48). For real-time PCR, amplification was performed using the SYBR green mix kit, and real-time fluorescence was detected by using the Applied Biosystems real-time PCR apparatus (Life Technology). The primers used for the reactions are listed in Table 2. The 16S rRNA was used as an internal control to normalize variation due to differences in reverse transcription efficiency. The 2^−ΔΔCT^ method was used to calculate fold change of the genes being tested (49). The PCR was performed as follows: 50°C, 2 min; 95°C, 2 min; and then 95°C, 15 s; 55°C, 15 s; 72°C, 30 s for 40 cycles. Each amplification reaction was performed in triplicate.

**TABLE 2:** Primers used in this study

Use and name of primer	Sequence (5′–3′)
Real-time qPCR	
16S-FA-F	gggtccccgtcaattccttt
16S-FA-R	tcggtgccgaagttaacaca
FA519-qPCR-F	gagtatctatgggaaaggtatttca
FA519-qPCR-R	aaattccgtcttcaacgtct
Mutant construction	
P1-FA519-up-For	ttaattgtgagtcatcaccc
P2-FA519-erm-Rev	atttattcctcctagttagtcaaacctacctctattctaaatac
P3-erm-For	tgactaactaggaggaataaatgacaaaaaagaaattgcccg
P4-erm-Rev	gattattccctccaggtactacgaaggatgaaatttttca
P5-FA519-erm-For	gtagtacctggagggaataatctgtagtgatgacaaatagaac
P6-FA519-dn-Rev	gtcttgaaacagatgcttttg
Overexpression	
FA519-pPROEXHTA-BamHI-F	aaaggatccgatgagaattgacaagggatatac ^*a*^
FA519-pPROEXHTA-SalI-R	gaagtcgacctattctccgtcttcctct ^*b*^
Protection Assays	
DNAJ-F	atggagaaaagagattattatg
DNAJ-R	ttaatctaatgcatctttaattt
RNA Polß-F	atgccacatccacatcctg
RNA Polß-R	ctaaaaatccagactgtcatc
FA1654-F	aacttggcggtaatgacatttaaattac
FA1654-R	ttatttcacgcttgagttaatgaacc

a Underlining indicates the BamHI restriction site.

b Underlining indicates the SalI restriction site.

### Construction of *F. alocis* mutant (Δ*FA519*::*ermF*).

*F. alocis* isogenic mutant was created by allelic-exchange mutagenesis using overlap extension PCR as described previously with some modifications (21). The primers used are shown in Table 2. Briefly, ∼600 bp flanking fragments of the gene *FA519* both upstream and downstream of the target genes were PCR amplified from chromosomal DNA of *F. alocis* ATCC 35896. The promoterless *ermF* cassette was amplified from the pVA2198 plasmid (50) with oligonucleotide primers that contain overlapping nucleotides for the upstream and downstream fragments of the gene, respectively. The upstream fragment, *ermF*, and downstream fragments were fused together and the purified fusion product was electroporated into *F. alocis* competent cells (see below). The electroporated cells were plated on BHI agar containing 5 μg/ml of erythromycin and incubated anaerobically at 37°C for 5 to 7 days. PCR and DNA sequencing were used to screen the colonies grown on erythromycin plates for the correct gene placement.

### Electroporation of *F. alocis*.

Electroporation of *F. alocis* was carried out as described previously (21). Briefly, 4 ml of BHI broth was inoculated with 1 ml of actively growing *F. alocis* culture and incubated at 37°C in the anaerobic chamber overnight. The cells from 5 ml culture (OD_600_ of ∼0.23) were harvested by centrifugation at 2,600 × *g* for 10 min at 4°C, washed, resuspended in 1 ml of ice-cold electroporation buffer (10% glycerol, 1 mM MgCl_2_), and incubated on ice for 20 min. Cells were centrifuged again; the pellet was washed with 1 ml of 10% glycerol and finally resuspended in 100 μl of 10% glycerol. A total of 1 to 4 μg of DNA was added to the 100 μl competent cells. The cell suspension was placed in a sterile electrode cuvette (0.2-cm gap) and then incubated on ice for 10 min. The cells were pulsed with a Bio-Rad gene pulser at 2.5 V and 600 Ω. The cuvettes were immediately placed inside the anaerobic chamber, and 500 μl of BHI broth was added to the *F. alocis* cells and incubated at 37°C for approximately 24 h. Cells were then plated on BHI agar plates containing 5 μg/ml erythromycin and incubated anaerobically at 37°C for 5 to 7 days.

### Cloning, expression, and purification of rFA519.

The gene encoding FA519 was PCR amplified using *F. alocis* ATCC 35896 genomic DNA and a pair of oligonucleotide primers (FA519-pPROEXHTA-BAMHI-F, FA519-pPROEXHTA-SalI; Table 2). The PCR product was then purified, digested with BamHI and SalI, and subsequently ligated into the plasmid pPROEX HTA that had been precut with BamHI and SalI restriction enzymes. The resulting ligation mix was transformed into the E. coli TOP10 and plated on LB agar plates containing 100 μg/ml of ampicillin. Bacterial colonies were screened by colony PCR to identify the positive clone, which was further verified by restriction enzyme digestion and DNA sequencing. The r-plasmid pPROEX HTA-*FA519* was then transformed into E. coli BL21(DE3) cells for expression.

A 1-liter culture of E. coli BL21 cells harboring the recombinant plasmid was grown at 37°C in LB medium containing 100 μg/ml of ampicillin until an OD_600_ of ∼0.6. FA519 expression was induced with 0.4 mM IPTG (isopropyl-β-d-thiogalactopyranoside) at 37°C for 4 h. Once expressed, the protein was purified under native conditions using Ni-nitrilotriacetic acid (Ni-NTA) resin according to the manufacturer’s instructions (Qiagen). The purified recombinant protein (rFA519) was resolved on SDS-PAGE gel. The pure protein fractions were subsequently dialyzed twice against 1 liter of dialysis buffer containing Tris buffer (50 mM [pH 7.8]) and 5% glycerol at 4°C and stored at −80°C in aliquots.

### Hydrogen peroxide (H_2_O_2_) sensitivity assay.

Sensitivity of *F. alocis* strains to H_2_O_2_ was determined as described previously with slight modifications (21). Briefly, bacterial cultures were grown overnight in BHI broth without cysteine at 37°C (OD_600_ of ∼0.22). The cultures were diluted in BHI without cysteine to a starting OD_600_ of 0.05 of which 12 ml of the bacterial culture was treated with 0.25 mM H_2_O_2_ and incubated at 37°C for 1 h. After incubation, H_2_O_2_-exposed bacterial cultures were serially diluted in phosphate-buffered saline (PBS) and appropriate dilutions were plated on BHI agar plates. Colonies were enumerated and CFU/ml was determined after 7 days of anaerobic incubation at 37°C. Percent survival was calculated as the ratio of the CFU/ml of H_2_O_2_-treated divided by the untreated input. Experiments were done in triplicate in three independent repeats.

### Sensitivity to nitric oxide.

NO was produced using the NO donor diethylamine (DEA) NONOate (Cayman Chemical, Ann Arbor, MI). *F. alocis* strains were grown overnight in BHI broth without cysteine at 37°C (OD_600_ of ∼0.22). The cultures were diluted in BHI without cysteine to a starting OD of 0.05 (0.05 OD_600_). Then, 12 ml of bacterial culture was treated with 9.6 μM NO and incubated at 37°C for 1 h. After incubation, NO-exposed bacterial cultures were serially diluted in phosphate-buffered saline (PBS) and appropriate dilutions were plated on BHI agar plates. Colonies were enumerated and CFU/ml was determined after 7 days of anaerobic incubation at 37°C. Percent survival was calculated as the ratio of the CFU/ml of NO-treated divided by the untreated input. Experiments were done in triplicate in three independent repeats.

### Biofilm assays.

*In vitro* biofilm assay was done as described previously with minor modifications (51). Briefly, *F. alocis* and P. gingivalis ATCC 33277 (*PG33277*) were grown overnight in BHI broth at 37°C (OD_600_ of ∼0.22 and OD_600_ of ∼1.0, respectively). Cultures were diluted to OD_600_ of ∼0.05 in BHI, and 200 μl of diluted culture was used to inoculate sterile, 96-well polystyrene plates (Greiner Bio-One) and incubated at 37°C inside the anaerobic chamber. For dual species biofilm, cultures were mixed 1:1 (vol/vol). After 48 h, wells containing the stationary-phase cells with similar OD_600_ were used to perform biofilm assay. Free-floating cells were aspirated and the attached biofilm growth was gently washed three times with 200 μl of PBS and subsequently stained with 100 μl of 0.5% crystal violet for 30 min. The unbound dye was completely removed by washing several times with PBS. For quantitative analysis of biofilm production, the wells were destained with 100 μl of ethanol/acetone mix (80:20) for 15 min. Optical density at 580 nm was measured using xMark microplate spectrophotometer (Bio-Rad). BHI broth was used as blank. The assay was done in quadruplicates in four independent experiments.

### Epithelial cell culture conditions.

Telomerase-immortalized gingival keratinocytes (TIGKs) were cultured in DermaLife K serum‐free keratinocyte culture medium (Lifeline Cell Technology) supplemented with 0.5 ng/ml transforming growth factor alpha (TGFα), 5 μg/ml insulin, 1 μM epinephrine, 5 μg/ml apo-transferrin, 100 ng/ml hydrocortisone, 0.4% bovine pituitary extract, and 6 mM l-glutamine at 37°C under 5% CO_2_. At about 70% confluence, monocultures were trypsinized and adjusted to approximately 1 × 10^3^ cells/ml, flowed by seeding 1 ml per well into 12-well plates (3.5 cm^2^ area; ThermoFisher), and further incubated for 48 h to reach semiconfluence (10^5^ cells per well).

### Cell adhesion and invasion assays.

The adhesion and invasion assays were performed using standard antibiotic protection assay as described previously with minor modifications (21). Briefly, overnight-grown *F. alocis* cultures (OD_600_ of ∼0.22) were used to infect TIGK cells (grown in 12-well plates [10^5^ cells per well]) with a multiplicity of infection (MOI) of 100 (∼10^7^ CFU/ml) for 1 h at 37°C. After 1 h of incubation, nonadherent bacteria were removed by washing the cells with PBS, and washed cells were detached with 0.05% trypsin and lysed by osmotic lysis in sterile distilled water and 0.025% Triton X-100. The appropriate dilutions were plated on BHI agar plates to enumerate the adherent bacteria. For the invasion assay, following a 1-h infection, the extracellular bacteria were killed by incubating the cells with 200 μg/ml of metronidazole for 1 h. The cells were then washed, detached, and lysed. Lysates were serially diluted and plated on BHI agar plates. Plates from both adhesion and invasion assays were incubated anaerobically at 37°C for 5 to 7 days, after which the colonies were counted and CFU/ml was calculated. The CFU of adherent and/or invaded bacteria for each strain was compared with input titer, and the percentages of adherent and invaded bacteria were determined. Each assay was done in triplicate in three independent repeats inside the anaerobic chamber.

### Zinc-binding assays.

Zinc binding by FA519 was determined using the Zn-binding dye 4-(2-pyridylazo)-resorcinol (PAR) as described previously, with slight modifications (52, 53). Free PAR exhibits a peak absorbance at approximately 410 nm, which shifts to 500 nm upon Zn binding. First, the metal standard solution (for the standard curve) or the protein solution (for the experimental data) is mixed with guanidine–HCl (4 M, final concentration) and incubated at room temperature for 1 to 3 min. A freshly prepared solution of PAR (final concentration 100 mM) is then mixed with the sample. The absorption spectra of PAR and PAR–metal complexes were determined from 350 to 600 nm. To determine if FA519 binds Zn, increasing concentrations (0 μM to 4 μM) of purified rFA519 were added to solutions containing 100 μM PAR and 25 μM Zn in 50 mM HEPES [pH 8.0]. Spectra were obtained for free PAR and PAR bound to Zn as controls, which were compared to the spectra obtained in the presence of rFA519. A decrease in the absorbance at 500 nm, together with an increase in the absorbance at 410 nm, indicates binding of Zn by rFA519. Spectra were also obtained for free PAR in the presence of rFA519. A standard curve (0 to 10 μM) was assayed with each series of experimental samples.

### DNA protection from oxidative damage.

The ability of FA519 to putatively bind to DNA and prevent DNA from Fenton-mediated DNA damage was assessed *in vitro* by monitoring DNA damage by hydroxyl radicals by observing the degradation of DNA in the presence of Fe(II), H_2_O_2_, and rFA519. This was carried out by the Fenton reaction as described previously (54) with slight modifications. Briefly, randomly chosen DNA fragments from *F. alocis* HMPREF0389_00093 (DNAJ), HMPREF0389_01735 (RNA polymerase β subunit), and HMPREF0389_01654 (FA1654) were amplified from *F. alocis* genomic DNA (primers are listed in Table 2) and purified. The pBR322 plasmid (4.361 kb) was obtained from New England Biolabs Inc., and 200 ng of DNA was used in all experiments. Plasmid DNA or DNA fragments were incubated with 10 μM rFA519 in the presence of FeSO4 (50 μM) in Tris buffer (20 mM [pH 7.8]) and double-distilled water for a final volume of 18 μl. The reaction was allowed to incubate at room temperature for 15 min. A total of 2 μl H_2_O_2_ (880 mM) was added to the appropriate reactions and allowed to further incubate for 30 min at 37°C. The presence or absence of rFA519 was used as the control. A total of 2 μl of 6× gel loading dye (New England Biolabs) was added to the mixture, and the entire reaction was loaded on a 1% agarose gel. The gel was stained with ethidium bromide after electrophoresis and visualized using UVP Chemstudio touch, 815 (Analytik Jena).

### MCO assay.

The antioxidant activity of FA519 was determined using the MCO assay with slight modifications (55). First, the reaction mixture containing Tris buffer (25 mM [pH 7.8]), 5 mM DTT, and 3 μM FeCl_3_ was incubated at room temperature for 30 min. rFA519 (2, 10, 15, 20, and 25 μM) was added to the reaction mix followed by incubation at 37°C for 2 h. The pBR322 plasmid DNA (250 ng) was added to the reaction mixture, followed by incubation for an additional 2 h. Plasmid damage was assessed following 1% agarose gel electrophoresis, ethidium bromide staining, and visualization using UVP Chemstudio touch, 815 (Analytik Jena).

### *In vitro* enzyme activity assay.

To investigate whether FA519 possesses peroxidase activity, the antioxidant activity of rFA519, reflected by the removal of H_2_O_2_, was assessed as described by Lim et al. (25), with slight modifications. Briefly, 200-μl reaction mixtures containing 50 mM HEPES (pH 7.0) and rFA519 (0.5, 5, and 10 μM) were preincubated with or without 5 mM dithiothreitol (DTT) or 5 mM glutathione (GSH) at room temperature for 10 min. After, 200 μM H_2_O_2_ was added and further incubated for 10 min. The reaction was stopped by addition of 10% (vol/vol) trichloroacetic acid. Then, 40 μl of 10 mM ammonium iron (II) sulfate [Fe(NH_4_)_2_(SO_4_)_2_] and 20 μl of 2.5 M potassium thiocyanate (KSCN) was added to react with the remaining H_2_O_2_ to produce the ferrithiocyanate complex measured by colorimetry at 475 nm (Bio-Rad xMark microplate spectrophotometer), the absorbance maximum of the complex. Control experiments included reactions with and without rFA519. Peroxidase activity was also confirmed using the ferrous xylenol orange method as described previously (53). Briefly, the assay was carried out in 40 mM potassium phosphate buffer (pH 7.0), 1 mM DTT, and 10 μM rFA519, and the reaction mixture was incubated at room temperature. After 10 min, 200 μM H_2_O_2_ was added to the reaction. At various time points (0, 2, 3, 4, 5, and 10 min), 10 μl of the assay was added to 200 μl xylenol orange solution (250 mM sulfuric acid, 1 M sorbitol, 2.5 mM ferrous ammonium sulfate, and 1.25 mM xylenol orange in H_2_O). The decrease in H_2_O_2_ levels was determined spectrophotometrically by measuring absorbance at 560 nm.

### Determination of peroxidase activity linked to thioredoxin.

The peroxidase activity linked to thioredoxin was assayed using the ferrithiocyanate system as described previously (55), with minor modifications. Briefly, the reaction was started by the addition of 1 mM H_2_O_2_ into the 50 μl of reaction mixture containing 10 μM rFA519, 2 mM NADPH, 12.5 μg/ml of E. coli thioredoxin (Trx), 12.5 μg/ml of E. coli thioredoxin reductase (TrxR), 1 mM EDTA, and 50 mM HEPES-NaOH [pH 7.0] and then incubated at 37°C. After, the reaction mixture was added to 10% trichloroacetic acid solution to stop the reaction, followed by the addition of 10 mM Fe(NH_4_)_2_(SO_4_)_2_ and 2.5 N KSCN to develop the complex, giving a purple color. The concentration of H_2_O_2_ was monitored by measurement of the decrease in absorbance at 475 nm. To determine peroxidase activity of rFA519 linked to NADPH oxidation, the reaction was started by the addition of 10 μM rFA519 to 50 mM HEPES-NaOH buffer (pH 7.0), containing 0.375 mM NADPH, 1 mM H_2_O_2_, 6.4 μM Trx, and 0.14 μM TrxR. The resulting oxidation of NADPH was directly followed by the decrease in absorbance at 340 nm. For controls, Trx, TrxR, rFA519, or H_2_O_2_ was omitted from the reaction mixtures.

### Oxidoreductase activity assay.

The insulin reduction assay, a reductase activity assay, is a common method used to determine whether a protein can function as an oxidoreductase, regardless of its function in the reducing or the oxidizing pathway *in vivo*. The ability of rFA519 to catalyze insulin reduction in the presence of DTT was determined as described previously (56). Briefly, reaction mixtures (200 μl) were prepared in 96-well plates containing 150 μM insulin and increasing concentrations of rFA519 in 0.1 M potassium phosphate buffer (pH 7.0) and 2 mM EDTA. Reactions were started by adding DTT to a final concentration of 1 mM. After thorough mixing, the optical density at 650 nm was recorded every 5 min. The noncatalyzed reduction of insulin by DTT was monitored in a control reaction without addition of catalyst.

### Data availability.

The RNA-Seq data were submitted to the Gene Expression Omnibus database (https://www.ncbi.nlm.nih.gov/geo, accession number: GSE181879).

## References

[1] Henry LG, McKenzie RM, Robles A, Fletcher HM. 2012. Oxidative stress resistance in *Porphyromonas gingivalis*. Future Microbiol 7:497–512. doi:10.2217/fmb.12.17. 22439726 PMC3397238

[2] Auten RL, Davis JM. 2009. Oxygen toxicity and reactive oxygen species: the devil is in the details. Pediatr Res 66:121–127. doi:10.1203/PDR.0b013e3181a9eafb. 19390491

[3] Chapple IL, Matthews JB. 2007. The role of reactive oxygen and antioxidant species in periodontal tissue destruction. Periodontol 2000 43:160–232. doi:10.1111/j.1600-0757.2006.00178.x. 17214840

[4] Chiu AV, Saigh MA, McCulloch CA, Glogauer M. 2017. The role of NrF2 in the regulation of periodontal health and disease. J Dent Res 96:975–983. doi:10.1177/0022034517715007. 28617616

[5] Ezraty B, Gennaris A, Barras F, Collet JF. 2017. Oxidative stress, protein damage and repair in bacteria. Nat Rev Microbiol 15:385–396. doi:10.1038/nrmicro.2017.26. 28420885

[6] Dahlen G, Leonhardt A. 2006. A new checkerboard panel for testing bacterial markers in periodontal disease. Oral Microbiol Immunol 21:6–11. doi:10.1111/j.1399-302X.2005.00243.x. 16390335

[7] Kumar PS, Leys EJ, Bryk JM, Martinez FJ, Moeschberger ML, Griffen AL. 2006. Changes in periodontal health status are associated with bacterial community shifts as assessed by quantitative 16S cloning and sequencing. J Clin Microbiol 44:3665–3673. doi:10.1128/JCM.00317-06. 17021095 PMC1594761

[8] Kumar PS, Griffen AL, Moeschberger ML, Leys EJ. 2005. Identification of candidate periodontal pathogens and beneficial species by quantitative 16S clonal analysis. J Clin Microbiol 43:3944–3955. doi:10.1128/JCM.43.8.3944-3955.2005. 16081935 PMC1233920

[9] Schlafer S, Riep B, Griffen AL, Petrich A, Hubner J, Berning M, Friedmann A, Gobel UB, Moter A. 2010. *Filifactor alocis*–involvement in periodontal biofilms. BMC Microbiol 10:66. doi:10.1186/1471-2180-10-66. 20193074 PMC2846919

[10] Ramesh A, Varghese S, Jayakumar ND, Malaiappan S. 2018. Comparative estimation of sulfiredoxin levels between chronic periodontitis and healthy patients - a case-control study. J Periodontol 89:1241–1248. doi:10.1002/JPER.17-0445. 30044495

[11] Hajishengallis G. 2015. Periodontitis: from microbial immune subversion to systemic inflammation. Nat Rev Immunol 15:30–44. doi:10.1038/nri3785. 25534621 PMC4276050

[12] Kumar PS, Griffen AL, Barton JA, Paster BJ, Moeschberger ML, Leys EJ. 2003. New bacterial species associated with chronic periodontitis. J Dent Res 82:338–344. doi:10.1177/154405910308200503. 12709498

[13] Wade WG. 2011. Has the use of molecular methods for the characterization of the human oral microbiome changed our understanding of the role of bacteria in the pathogenesis of periodontal disease? J Clin Periodontol 38 Suppl 11:7–16. doi:10.1111/j.1600-051X.2010.01679.x. 21323699

[14] Bingham CO, Moni M. 2013. Periodontal disease and rheumatoid arthritis: the evidence accumulates for complex pathobiologic interactions. Curr Opin Rheumatol 25:345–353. doi:10.1097/BOR.0b013e32835fb8ec. 23455329 PMC4495574

[15] Kumar PS. 2013. Oral microbiota and systemic disease. Anaerobe 24:90–93. doi:10.1016/j.anaerobe.2013.09.010. 24128801

[16] Maddi A, Scannapieco FA. 2013. Oral biofilms, oral and periodontal infections, and systemic disease. Am J Dent 26:249–254. 24479275

[17] Poole S, Singhrao SK, Kesavalu L, Curtis MA, Crean S. 2013. Determining the presence of periodontopathic virulence factors in short-term postmortem Alzheimer’s disease brain tissue. J Alzheimers Dis 36:665–677. doi:10.3233/JAD-121918. 23666172

[18] Aruni AW, Roy F, Fletcher HM. 2011. *Filifactor alocis* has virulence attributes that can enhance its persistence under oxidative stress conditions and mediate invasion of epithelial cells by *Porphyromonas gingivalis*. Infect Immun 79:3872–3886. doi:10.1128/IAI.05631-11. 21825062 PMC3187275

[19] Aruni W, Chioma O, Fletcher HM. 2014. *Filifactor alocis*: the newly discovered kid on the block with special talents. J Dent Res 93:725–732. doi:10.1177/0022034514538283. 24898946 PMC4126222

[20] Aruni AW, Mishra A, Dou Y, Chioma O, Hamilton BN, Fletcher HM. 2015. *Filifactor alocis*–a new emerging periodontal pathogen. Microbes Infect 17:517–530. doi:10.1016/j.micinf.2015.03.011. 25841800 PMC4485945

[21] Mishra A, Aja E, Fletcher HM. 2020. Role of superoxide reductase FA796 in oxidative stress resistance in *Filifactor alocis*. Sci Rep 10:9178. doi:10.1038/s41598-020-65806-3. 32513978 PMC7280497

[22] Aruni AW, Roy F, Sandberg L, Fletcher HM. 2012. Proteome variation among *Filifactor alocis* strains. Proteomics 12:3343–3364. doi:10.1002/pmic.201200211. 23008013 PMC4514522

[23] Ortiz de Orue Lucana D, Wedderhoff I, Groves MR. 2012. ROS-mediated signalling in bacteria: zinc-containing Cys-X-X-Cys redox centres and iron-based oxidative stress. J Signal Transduct 2012:605905. doi:10.1155/2012/605905. 21977318 PMC3184428

[24] Aruni AW, Zhang K, Dou Y, Fletcher H. 2014. Proteome analysis of coinfection of epithelial cells with *Filifactor alocis* and *Porphyromonas gingivalis* shows modulation of pathogen and host regulatory pathways. Infect Immun 82:3261–3274. doi:10.1128/IAI.01727-14. 24866790 PMC4136196

[25] Lim YS, Cha MK, Kim HK, Uhm TB, Park JW, Kim K, Kim IH. 1993. Removals of hydrogen peroxide and hydroxyl radical by thiol-specific antioxidant protein as a possible role *in vivo*. Biochem Biophys Res Commun 192:273–280. doi:10.1006/bbrc.1993.1410. 8386507

[26] Sevier CS, Kadokura H, Tam VC, Beckwith J, Fass D, Kaiser CA. 2005. The prokaryotic enzyme DsbB may share key structural features with eukaryotic disulfide bond forming oxidoreductases. Protein Sci 14:1630–1642. doi:10.1110/ps.051355705. 15930008 PMC2253379

[27] Ladenstein R, Ren B. 2006. Protein disulfides and protein disulfide oxidoreductases in hyperthermophiles. FEBS J 273:4170–4185. doi:10.1111/j.1742-4658.2006.05421.x. 16930136

[28] Eke PI, Dye BA, Wei L, Slade GD, Thornton-Evans GO, Borgnakke WS, Taylor GW, Page RC, Beck JD, Genco RJ. 2015. Update on prevalence of periodontitis in adults in the United States: NHANES 2009 to 2012. J Periodontol 86:611–622. doi:10.1902/jop.2015.140520. 25688694 PMC4460825

[29] Sczepanik FSC, Grossi ML, Casati M, Goldberg M, Glogauer M, Fine N, Tenenbaum HC. 2020. Periodontitis is an inflammatory disease of oxidative stress: we should treat it that way. Periodontol 2000 84:45–68. doi:10.1111/prd.12342. 32844417

[30] Aja E, Mangar M, Fletcher HM, Mishra A. 2021. *Filifactor alocis*: recent insights and advances. J Dent Res 100:790–797. doi:10.1177/00220345211000656. 33719654 PMC8261852

[31] Krishna SS, Majumdar I, Grishin NV. 2003. Structural classification of zinc fingers: survey and summary. Nucleic Acids Res 31:532–550. doi:10.1093/nar/gkg161. 12527760 PMC140525

[32] Jakob U, Eser M, Bardwell JC. 2000. Redox switch of Hsp33 has a novel zinc-binding motif. J Biol Chem 275:38302–38310. doi:10.1074/jbc.M005957200. 10976105

[33] Laity JH, Lee BM, Wright PE. 2001. Zinc finger proteins: new insights into structural and functional diversity. Curr Opin Struct Biol 11:39–46. doi:10.1016/s0959-440x(00)00167-6. 11179890

[34] Liu X, Ramsey MM, Chen X, Koley D, Whiteley M, Bard AJ. 2011. Real-time mapping of a hydrogen peroxide concentration profile across a polymicrobial bacterial biofilm using scanning electrochemical microscopy. Proc Natl Acad Sci USA 108:2668–2673. doi:10.1073/pnas.1018391108. 21282623 PMC3041060

[35] Huergo LF, Rahman H, Ibrahimovic A, Day CJ, Korolik V. 2013. *Campylobacter jejuni* Dps protein binds DNA in the presence of iron or hydrogen peroxide. J Bacteriol 195:1970–1978. doi:10.1128/JB.00059-13. 23435977 PMC3624597

[36] Martin JL. 1995. Thioredoxin–a fold for all reasons. Structure 3:245–250. doi:10.1016/S0969-2126(01)00154-X. 7788290

[37] Wouters MA, Fan SW, Haworth NL. 2010. Disulfides as redox switches: from molecular mechanisms to functional significance. Antioxid Redox Signal 12:53–91. doi:10.1089/ars.2009.2510. 19634988

[38] Holmgren A. 1985. Thioredoxin. Annu Rev Biochem 54:237–271. doi:10.1146/annurev.bi.54.070185.001321. 3896121

[39] Holmgren A. 1979. Thioredoxin catalyzes the reduction of insulin disulfides by dithiothreitol and dihydrolipoamide. J Biol Chem 254:9627–9632. doi:10.1016/S0021-9258(19)83562-7. 385588

[40] Hanschmann EM, Godoy JR, Berndt C, Hudemann C, Lillig CH. 2013. Thioredoxins, glutaredoxins, and peroxiredoxins–molecular mechanisms and health significance: from cofactors to antioxidants to redox signaling. Antioxid Redox Signal 19:1539–1605. doi:10.1089/ars.2012.4599. 23397885 PMC3797455

[41] Khairnar NP, Joe MH, Misra HS, Lim SY, Kim DH. 2013. FrnE, a cadmium-inducible protein in *Deinococcus radiodurans*, is characterized as a disulfide isomerase chaperone *in vitro* and for its role in oxidative stress tolerance *in vivo*. J Bacteriol 195:2880–2886. doi:10.1128/JB.01503-12. 23603741 PMC3697258

[42] Gonzalez D, Alamos P, Rivero M, Orellana O, Norambuena J, Chavez R, Levican G. 2020. Deciphering the role of multiple thioredoxin fold proteins of *Leptospirillum sp*. in oxidative stress tolerance. Int J Mol Sci 21:1880. doi:10.3390/ijms21051880. 32164170 PMC7084401

[43] Klomsiri C, Karplus PA, Poole LB. 2011. Cysteine-based redox switches in enzymes. Antioxid Redox Signal 14:1065–1077. doi:10.1089/ars.2010.3376. 20799881 PMC3064533

[44] Cremers CM, Jakob U. 2013. Oxidant sensing by reversible disulfide bond formation. J Biol Chem 288:26489–26496. doi:10.1074/jbc.R113.462929. 23861395 PMC3772196

[45] Broden NJ, Flury S, King AN, Schroeder BW, Coe GD, Faulkner MJ. 2016. Insights into the function of a second, nonclassical Ahp peroxidase, AhpA, in oxidative stress resistance in *Bacillus subtilis*. J Bacteriol 198:1044–1057. doi:10.1128/JB.00679-15. 26787766 PMC4800881

[46] Fomenko DE, Koc A, Agisheva N, Jacobsen M, Kaya A, Malinouski M, Rutherford JC, Siu KL, Jin DY, Winge DR, Gladyshev VN. 2011. Thiol peroxidases mediate specific genome-wide regulation of gene expression in response to hydrogen peroxide. Proc Natl Acad Sci USA 108:2729–2734. doi:10.1073/pnas.1010721108. 21282621 PMC3041109

[47] Dou Y, Rutanhira H, Chen X, Mishra A, Wang C, Fletcher HM. 2018. Role of extracytoplasmic function sigma factor PG1660 (RpoE) in the oxidative stress resistance regulatory network of *Porphyromonas gingivalis*. Mol Oral Microbiol 33:89–104. doi:10.1111/omi.12204. 29059500 PMC5823243

[48] Dou Y, Rutanhira H, Schormann N, Deivanayagam C, Fletcher HM. 2021. PG1659 functions as anti-sigma factor to extracytoplasmic function sigma factor RpoE in *Porphyromonas gingivalis* W83. Mol Oral Microbiol 36:80–91. doi:10.1111/omi.12329. 33377315 PMC7940587

[49] Livak KJ, Schmittgen TD. 2001. Analysis of relative gene expression data using real-time quantitative PCR and the 2(-Delta Delta C(T)) method. Methods 25:402–408. doi:10.1006/meth.2001.1262. 11846609

[50] Fletcher HM, Schenkein HA, Morgan RM, Bailey KA, Berry CR, Macrina FL. 1995. Virulence of a *Porphyromonas gingivalis* W83 mutant defective in the *prtH* gene. Infect Immun 63:1521–1528. doi:10.1128/iai.63.4.1521-1528.1995. 7890419 PMC173184

[51] Mishra A, Wu C, Yang J, Cisar JO, Das A, Ton-That H. 2010. The *Actinomyces oris* type 2 fimbrial shaft FimA mediates co-aggregation with oral streptococci, adherence to red blood cells and biofilm development. Mol Microbiol 77:841–854. doi:10.1111/j.1365-2958.2010.07252.x. 20545853 PMC2946971

[52] Hood MI, Mortensen BL, Moore JL, Zhang Y, Kehl-Fie TE, Sugitani N, Chazin WJ, Caprioli RM, Skaar EP. 2012. Identification of an *Acinetobacter baumannii* zinc acquisition system that facilitates resistance to calprotectin-mediated zinc sequestration. PLoS Pathog 8:e1003068. doi:10.1371/journal.ppat.1003068. 23236280 PMC3516566

[53] Konig J, Galliardt H, Jutte P, Schaper S, Dittmann L, Dietz KJ. 2013. The conformational bases for the two functionalities of 2-cysteine peroxiredoxins as peroxidase and chaperone. J Exp Bot 64:3483–3497. doi:10.1093/jxb/ert184. 23828546 PMC3733160

[54] Henry LG, Aruni W, Sandberg L, Fletcher HM. 2013. Protective role of the PG1036-PG1037-PG1038 operon in oxidative stress in *Porphyromonas gingivalis* W83. PLoS One 8:e69645. doi:10.1371/journal.pone.0069645. 23990885 PMC3747172

[55] Zhang H, Wang Z, Huang J, Cao J, Zhou Y, Zhou J. 2020. A novel thioredoxin-dependent peroxiredoxin (TPx-Q) plays an important role in defense against oxidative stress and is a possible drug target in *Babesia microti*. Front Vet Sci 7:76. doi:10.3389/fvets.2020.00076. 32133382 PMC7040034

[56] Lafaye C, Iwema T, Carpentier P, Jullian-Binard C, Kroll JS, Collet JF, Serre L. 2009. Biochemical and structural study of the homologues of the thiol-disulfide oxidoreductase DsbA in *Neisseria meningitidis*. J Mol Biol 392:952–966. doi:10.1016/j.jmb.2009.07.056. 19631659

[57] Mishra A, Roy F, Dou Y, Zhang K, Tang H, Fletcher HM. 2018. Role of acetyltransferase PG1842 in gingipain biogenesis in *Porphyromonas gingivalis*. J Bacteriol 200. doi:10.1128/JB.00385-18. PMC6256024 30249709

